# A retrospective cohort study of H-type hypertension and its influence on the prognostic effect in patients with non-dialysis CKD

**DOI:** 10.3389/fnut.2025.1554663

**Published:** 2025-03-25

**Authors:** Xiaoyu Cai, Menglei Ju, Xinying Jiang, Shengnan Ge, Yuzhang Han, Shumin Lin, Hui Peng, Man Li, Cheng Wang

**Affiliations:** ^1^Division of Nephrology, Department of Medicine, The Fifth Affiliated Hospital of Sun Yat-Sen University, Zhuhai, Guangdong, China; ^2^Division of Nephrology, Department of Medicine, The Third Affiliated Hospital of Sun Yat-Sen University, Guangzhou, Guangdong, China; ^3^Guangdong Provincial Key Laboratory of Biomedical Imaging, The Fifth Affiliated Hospital of Sun Yat-Sen University, Zhuhai, Guangdong, China

**Keywords:** CKD, hyperhomocysteinemia, H-type hypertension, prognosis, MACCEs

## Abstract

**Background:**

The study aimed to investigate the impact of coexistence of hyperhomocysteinemia (HHcy) and hypertension (HTN), referred to as H-type hypertension on kidney outcomes and major adverse cardiovascular and cerebrovascular events (MACCEs) in patients with non-dialysis chronic kidney disease (CKD).

**Methods:**

This retrospective study enrolled 2,558 non-dialysis CKD patients admitted to two medical centers in China between 2010 and 2022. The participants were divided into four groups according to baseline blood pressure and homocysteine levels: (1) normotension with normohomocysteinemia; (2) normotension with HHcy; (3) hypertension with normohomocysteinemia; and (4) H-type hypertension. Cox regression model was applied to assess the relationship between these groups and renal outcomes/MACCEs. Mediation analysis was performed to assess the influence of HHcy on the link between hypertension and the outcomes.

**Results:**

Three hundred and eighty renal endpoint events and 211 MACCEs were recorded. The H-type hypertension group demonstrated higher incidence of renal events (age-adjusted incidence: 83.71/1,000 person-years vs. 24.50/1,000 person-years) and MACCEs (age-adjusted incidence: 41.28/1,000 person-years vs. 17.21/1,000 person-years) compared to the normotension with normohomocysteinemia group. After adjusting for confounders, H-type hypertension independently elevated the risk of kidney outcomes by 312% (HR = 4.12, 95% CI: 2.66–6.37) and MACCEs by 127% (HR = 2.27, 95% CI: 1.28–4.02). No statistically significant mediated effect of HHcy on the relationship between hypertension and renal outcomes or MACCEs was observed.

**Conclusion:**

H-type hypertension is associated with renal deterioration and cardiovascular events in non-dialysis CKD patients, early detections of H-type hypertension are essential to enhancing the prognosis for CKD patients.

## 1 Introduction

Chronic kidney disease (CKD) is a prevalent chronic medical condition, and its burden on healthcare systems has intensified with the aging population. It is estimated that CKD affects 9.1% (8.5–9.8%) of the global adults (1), with over 100 million cases in China (2). Cardiovascular disease (CVD) remains the primary cause of poor prognosis among CKD patients (3). The elevated incidence of traditional and non-traditional cardiovascular risk factors contributes to the development of CVD in CKD patients. Previous studies have linked age, hypertension (HTN), diabetes, and dyslipidemia with renal deterioration (4). Furthermore, research into the pathogenesis of atherosclerotic cardiovascular events has highlighted hyperhomocysteinemia (HHcy) as a non-traditional risk factor for CVD (5), but the effect of HHcy in CKD patients was unclear. Identifying risk factors beyond hypertension is crucial to determining high-risk groups for adverse CKD outcomes.

As a sulfur-containing amino acid, plasma homocysteine (Hcy) levels exceeding 15 μmol/L are classified as HHcy. In China, a country without folate fortification policy and with differences in dietary patterns, the prevalence of HHcy in CKD patients reaches 52.78% (6), notably higher than in general population, and increases with advancing CKD stage. A prospective study by Ninomiya demonstrated that individuals with plasma Hcy levels in the highest tertile had a higher incidence of CKD compared to those in the lowest tertile (7). Previous researches have shown that HHcy linked to an elevated risk of cardiovascular atherosclerosis (8). In CKD patients, HHcy has been implicated in renal arteriosclerosis and declining glomerular filtration rates (GFR) (9). In addition to its role in renal outcomes, HHcy has been implicated in the pathogenesis of cerebrovascular events. For example, a recent retrospective cohort study demonstrated that elevated plasma Hcy levels were associated with an increased risk of ischemic stroke in hypertensive patients with obstructive sleep apnea (10). Furthermore, elevated serum homocysteine levels have been identified as a marker of CVD in individuals with end-stage renal disease (ESRD) or stable chronic kidney transplants (11, 12). However, the relationship between HHcy and CVD in CKD patients remains controversial, as studies by Suliman (13) and Kalantar-Zadeh (14) have reported conflicting findings, showing an inverse relationship between Hcy and cardiovascular mortality in ESRD patients.

The co-occurrence of hypertension and HHcy, known as H-type hypertension (HTH), has been observed in 44.14% of CKD patients (15). Compared to isolated HHcy or hypertension, patients with H-type hypertension were at a significantly greater risk of adverse cerebrovascular and cardiovascular events (16). Our previous study has demonstrated a link between H-type hypertension and carotid intima-media thickening, left ventricular hypertrophy, and elevated proteinuria levels in CKD patients (15). However, most of the evidence on the combined effect of homocysteine and hypertension comes from cross-sectional studies, primarily focusing on western populations, with limited prognostic data on CKD patients with H-type hypertension in China. Given the high incidence of CVD and the association between H-type hypertension and various organ damage in CKD patients, further investigation into the long-term prognosis is warranted. In this study, we enrolled non-dialysis CKD patients, measured baseline plasma Hcy levels, and evaluated the prognostic impact of H-type hypertension on renal outcomes and cardiovascular events in CKD patients.

## 2 Methods

### 2.1 Study design and participants

This retrospective study was conducted in the nephrology department of two large tertiary hospitals in Guangdong Province, China. The inclusion criteria comprised CKD patients diagnosed between August 2010 and December 2022 who met the following conditions: (i) diagnosis of non-dialysis CKD as per the Kidney Disease: Improving Global Outcomes (KDIGO) guidelines and no history of kidney transplantation; and (ii) aged 18–75 years. Exclusion criteria included pregnancy, HIV infection, malignancy, acute kidney injury (defined as a ≥ 30% reduction in estimated glomerular filtration rate [eGFR] within 3 months), patients undergoing high-dose corticosteroid therapy (≥0.5 mg/kg/d), and those with a history of severe cardiovascular conditions (myocardial infarction, atrial fibrillation, heart failure, stroke). Patients with incomplete or invalid follow-up data (i.e., those who reached endpoints or were lost to follow-up within the first 6 months) were also excluded. All patients meeting the inclusion criteria were consecutively enrolled, without stratification or matching, to reflect the characteristics of a real-world clinical population. The study received approval from the local ethics committees. Informed consent was obtained from all participants in compliance with the Declaration of Helsinki.

### 2.2 Data collection

Clinical data were collected at the first admission within 48 h from medical records, including demographics (age, sex, smoking, alcohol consumption, body mass index [BMI]), primary glomerulonephritis, comorbidities (identified by prior International Classification of Diseases [ICD] code without time limit), medications (antihypertensive drugs and folate intake), clinic blood pressure, and laboratory parameters (homocysteine, hemoglobin, serum albumin, urea, creatinine, uric acid, calcium-phosphorus product, fasting glucose, triglycerides, total cholesterol, high-density lipoprotein cholesterol [HDL-C], low-density lipoprotein cholesterol [LDL-C], intact parathyroid hormone [iPTH], 24-h proteinuria). Diabetes mellitus was defined according to pre-existing diagnosis documented in electronic health records (EHRs) using ICD-10-CM codes: E10, E11, E12, E13, E14, G59.0, G63.2, H28.0, H36.0, M14.2, N08.3, O24, P70.2, T38.3, Y42.3, and Z88.825. Antihypertensive medications included pre-admission drugs or those prescribed during hospitalization as clinically indicated, which included calcium channel blockers, angiotensin-converting enzyme inhibitors, angiotensin receptor blockers, α-blockers, and β-blockers. Folate was confirmed through either EHR documentation or self-reported medication histories prior to study enrollment or those prescribed during hospitalization as clinically indicated. Blood pressure measurement was conducted by trained nurses using validated oscillometric device with appropriately sized cuffs. Blood pressure was recorded on three separate occasions, spaced 1–2 min apart, and the average was calculated for analysis. Hyperhomocysteinemia was characterized by plasma Hcy concentration >15 μmol/L (15). Hypertension was characterized by blood pressure ≥140/90 mmHg. Based on blood pressure and plasma homocysteine levels, the cohort was divided into four groups: (1) normotension (NT) with normohomocysteinemia (NHcy); (2) normotension with HHcy; (3) hypertension with normohomocysteinemia; and (4) H-type hypertension, characterized by both hypertension and hyperhomocysteinemia.

### 2.3 Outcome ascertainment

The primary clinical endpoint was renal outcomes, which included a ≥50% decline in baseline eGFR, a doubling of serum creatinine, or the commencement of renal replacement therapy (peritoneal dialysis, hemodialysis, or kidney transplantation). Major adverse cardiovascular and cerebrovascular events encompassed cardiovascular mortality, non-fatal acute coronary syndrome (unstable angina and myocardial infarction), non-fatal stroke (including hemorrhagic and ischemic), new-onset heart failure, vascular reconstruction, and peripheral vascular disease, as outlined in previous research (17). Deaths were verified through medical records and family reports. Follow-up occurred every 6 months via telephone interviews or regular clinic visits, and follow-up duration was determined from the initial admission date to the occurrence of any endpoint. For individuals not reaching an endpoint, follow-up was calculated until the most recent visit before May 2024.

### 2.4 Statistical analysis

The Shapiro–Wilk test was used to evaluate the normality of the data. Continuous variables followed a normal distribution were expressed as mean ± standard deviation (SD) and analyzed through the Student’s t-test. For continuous variables that were not normally distributed, results were presented as median along with the 25th and 75th interquartile ranges (IQR), with the Mann–Whitney U test employed for between-group comparisons. Differences in categorical variables were assessed using the Chi-square test or Fisher’s exact test. Multiple imputation (MI) was applied to address missing data when the proportion of missing values was below 20%; variables with a missing rate exceeding 20% were excluded. Little’s test was used to assess whether the missing data were missing completely at random (MCAR). The comparison between pre- and post-interpolation was assessed using the Wilcoxon rank-sum test. Supplementary Table 1 showed the missingness report of our study, and we observed a consistent data distribution pattern both pre- and post-interpolation. Incidence rates, both crude and adjusted for age or sex, were calculated per 1,000 person-years. Kaplan–Meier survival curves and log-rank tests were used to analyze the cumulative risk differences among four groups, evaluating the impact of hypertension and hyperhomocysteinemia on prognosis. Directed acyclic graph (DAG) was employed to illustrate the relationship between outcomes and groups (Supplementary Figure 1). Multicollinearity was assessed using the variance inflation factor (VIF). Subsequently, univariate cox analysis was performed, and variables with *p*-values <0.05 and clinical relevance were selected for the multivariate cox regression model. Schoenfeld residuals were utilized to evaluate the proportional hazards assumption, and covariates that violated this assumption were subsequently modeled as time-dependent covariates. Hazard ratios (HR) and 95% confidence intervals (CI) were determined through cox regression models, using normal homocysteine level and blood pressure as reference group. Covariates in the model included sex, age, BMI, smoking, alcohol consumption, diabetes, 24-h proteinuria, hemoglobin, albumin, calcium-phosphate product, creatinine, uric acid, low-density lipoprotein cholesterol, high-density lipoprotein cholesterol, and iPTH. Separate univariate and multivariate cox regression analyses were conducted for populations with hypertension and normotension using homocysteine levels (per 1 SD) as continuous predictor. Additionally, sensitivity analysis-multivariate cox regression analysis of end point events according to Hyperhomocysteinemia/Hypertension groups was conducted in the complete case dataset.

Subgroup analyses were performed based on sex, diabetes, smoking, alcohol consumption, BMI (categorized as <24 kg/m^2^, 24–28 kg/m^2^, or ≥28 kg/m^2^), antihypertensive drugs or folate use, and eGFR (categorized as >60 mL/min/1.73 m^2^ or ≤60 mL/min/1.73 m^2^) to determine if demographic characteristics, lifestyle behaviors, and health conditions of CKD patients influenced the relationship between predictors and clinical outcomes. Interaction terms for the predictors and stratified variables were incorporated into the model, and the likelihood ratio test was utilized to evaluate the statistical significance of these interactions. The Benjamini–Hochberg procedure was applied to control the false discovery rate (FDR) in multiple hypothesis testing.

Mediation analysis was conducted to calculate the proportion of the effect of hypertension on outcomes mediated by hyperhomocysteinemia by comparing models with and without the proposed mediator (18). We performed 500 bootstrap resamples to compute bias-corrected 95% confidence intervals (CIs) for mediated proportion by the R package “mediation.”

To address potential selection bias and strengthen the robustness of the retrospective study, propensity score matching (PSM) analysis was conducted using a caliper of 0.05. “Nearest neighbor” matching model was performed to compare H-type hypertension with non-H-type hypertension and HHcy/HTN with NHcy/HTN groups in 1:1 ratio. Covariates for matching included sex, age, BMI, diabetes, smoking status, alcohol consumption, 24-h proteinuria, hemoglobin, albumin, calcium-phosphorus product, uric acid, low-density lipoprotein cholesterol, high-density lipoprotein cholesterol, and iPTH. Subsequently, cox regression models were utilized to evaluate the risks of adverse renal outcomes and MACCEs across the matched groups.

All statistical tests were two-tailed, with significance set at *p* < 0.05. All statistical analyses were conducted using RStudio software (version 1.1.423) and Prism 9 (GraphPad Software).

## 3 Results

### 3.1 Baseline characteristics of participants

**Figure 1:**
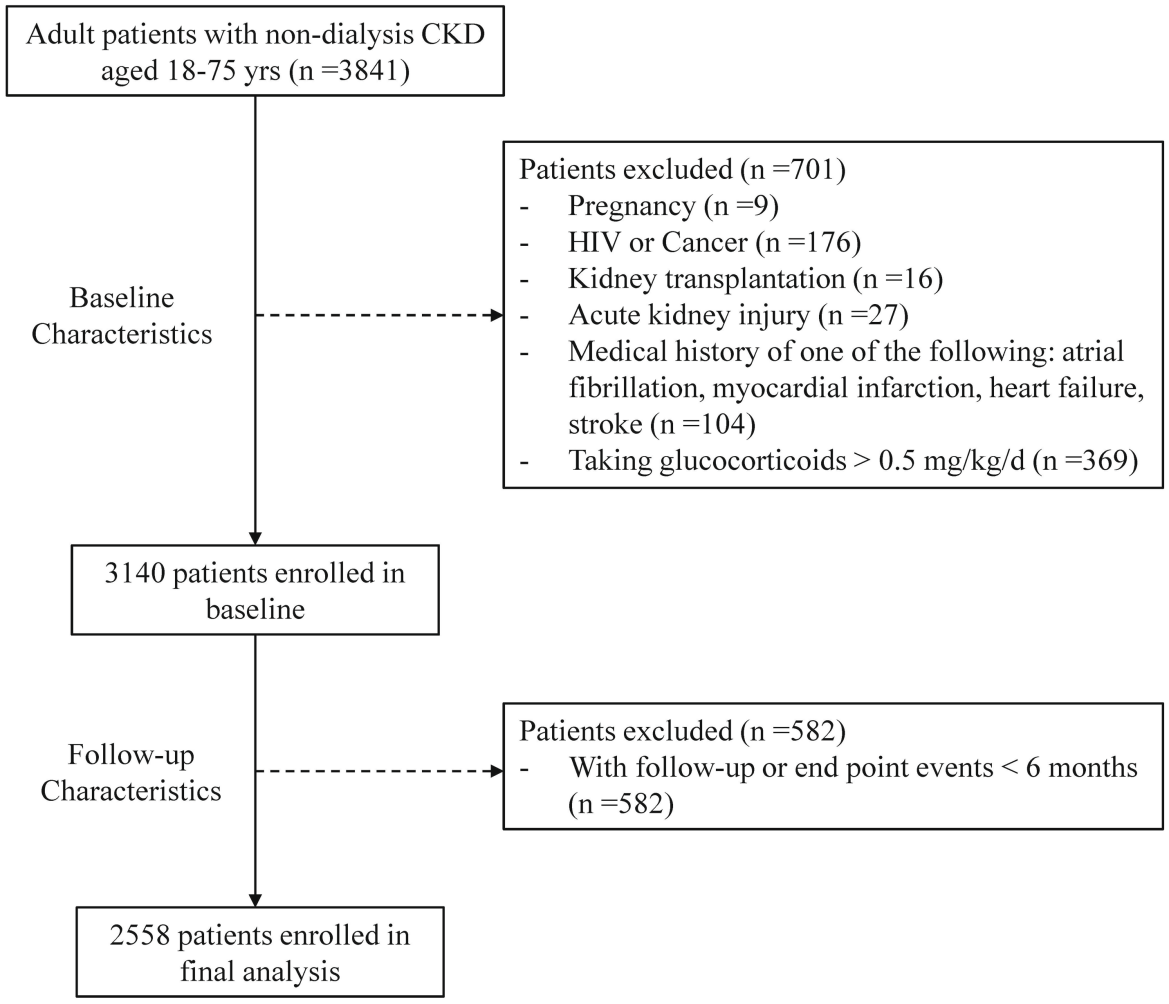
Enrollment of study population. CKD, chronic kidney disease; HIV, human immunodeficiency virus; AKI, acute kidney injury.

Of the 3,841 registered patients, 701 were excluded based on baseline characteristics, and 582 were removed due to lack of follow-up data or events occurring within 6 months. Consequently, 2,558 patients were enrolled in the analysis (Figure 1). Table 1 showed the baseline characteristics of participants categorized by the presence of hyperhomocysteinemia and hypertension. The average age was 47.78 years, with 57.2% being male. A total of 580 patients (22.7%) had diabetes, 29.1% smoked, and 24% reported alcohol consumption.

**Table 1:** Baseline characteristics of CKD patients with different blood pressure status and plasma homocysteine level.

	Total (*n* = 2,558)	Normotension and normohomocysteinemia (*n* = 620)	Normotension and hyperhomocysteinemia (*n* = 283)	Hypertension and normohomocysteinemia (*n* = 756)	H-type hypertension (*n* = 899)	*p* value
Sex: male (%)	1,463 (57.2)	264 (42.6)	207 (73.1)	390 (51.6)	602 (67.0)	<0.001
Age, yrs. (mean [SD])	47.78 (13.91)	40.84 (12.68)	46.33 (14.34)	49.03 (13.38)	51.96 (13.12)	<0.001
Course, mo (median [IQR])	8.00 [1.00, 36.00]	6.00 [1.00, 36.00]	7.00 [1.00, 36.00]	6.00 [1.00, 36.00]	12.00 [1.00, 36.00]	0.024
Composition of nephropathy						<0.001
Primary glomerulonephritis (%)	711 (27.8)	171 (27.6)	79 (27.9)	178 (23.5)	283 (31.5)	
IgA nephropathy (%)	512 (20.0)	161 (26.0)	63 (22.3)	143 (18.9)	145 (16.1)	
Membranous nephropathy (%)	188 (7.3)	53 (8.5)	14 (4.9)	96 (12.7)	25 (2.8)	
Minimal Change Disease (%)	84 (3.3)	39 (6.3)	9 (3.2)	28 (3.7)	8 (0.9)	
Focal Segmental Glomerulosclerosis (%)	59 (2.3)	19 (3.1)	4 (1.4)	18 (2.4)	18 (2.0)	
Diabetic nephropathy (%)	279 (10.9)	21 (3.4)	18 (6.4)	98 (13.0)	142 (15.8)	
Hypertensive nephropathy (%)	185 (7.2)	7 (1.1)	3 (1.1)	66 (8.7)	109 (12.1)	
Hyperuric acid nephropathy (%)	181 (7.1)	52 (8.4)	42 (14.8)	28 (3.7)	59 (6.6)	
Lupus nephritis (%)	57 (2.2)	18 (2.9)	7 (2.5)	17 (2.2)	15 (1.7)	
Polycystic Kidney Disease (%)	48 (1.9)	4 (0.6)	3 (1.1)	13 (1.7)	28 (3.1)	
Obstructive Nephropathy (%)	52 (2.0)	10 (1.6)	8 (2.8)	14 (1.9)	20 (2.2)	
Diabetes mellitus (%)	580 (22.7)	55 (8.9)	51 (18.0)	219 (29.0)	255 (28.4)	<0.001
Smoke (%)	745 (29.1)	115 (18.5)	102 (36.0)	194 (25.7)	334 (37.2)	<0.001
Alcohol (%)	613 (24.0)	114 (18.4)	65 (23.0)	186 (24.6)	248 (27.6)	0.001
Antihypertensive drugs						
Angiotensin-converting enzyme inhibitors (%)	113 (4.4)	5 (0.8)	1 (0.4)	71 (9.4)	36 (4.0)	<0.001
Angiotensin receptor blockers (%)	870 (34.0)	166 (26.8)	88 (31.1)	321 (42.5)	295 (32.8)	<0.001
Calcium channel blockers (%)	1,065 (41.6)	64 (10.3)	29 (10.2)	418 (55.3)	554 (61.6)	<0.001
β-blockers (%)	423 (16.5)	13 (2.1)	9 (3.2)	171 (22.6)	230 (25.6)	<0.001
α-blockers (%)	169 (6.6)	6 (1.0)	7 (2.5)	56 (7.4)	100 (11.1)	<0.001
Folate Tablets (%)	191 (7.5)	21 (3.4)	20 (7.1)	44 (5.8)	106 (11.8)	<0.001
BMI, kg/m^2^ (median [IQR])	23.94 [21.53, 26.44]	22.56 [20.15, 25.61]	23.53 [21.12, 25.36]	24.34 [22.08, 26.83]	24.53 [22.23, 26.99]	<0.001
Clinic SBP, mm Hg (mean (SD))	137.04 (23.63)	119.76 (15.48)	124.88 (19.68)	142.31 (21.18)	148.36 (22.84)	<0.001
Clinic DBP, mm Hg (mean (SD))	86.11 (14.55)	78.57 (10.31)	79.76 (11.89)	88.76 (13.78)	91.08 (15.60)	<0.001
Proteinuria, g/24 h (median [IQR])	0.89 [0.22, 2.52]	0.55 [0.13, 1.74]	0.55 [0.15, 1.99]	0.82 [0.21, 2.72]	1.30 [0.42, 2.99]	<0.001
Hemoglobin, g/L (mean (SD))	125.14 (25.18)	131.20 (21.66)	124.30 (25.41)	129.45 (24.06)	117.60 (26.34)	<0.001
Albumin, g/L (mean (SD))	37.63 (7.25)	37.69 (7.93)	38.80 (6.46)	37.00 (7.86)	37.75 (6.37)	0.004
Calcium*phosphate, mg^2^/dL^2^ (median [IQR])	2.41 [2.09, 2.90]	2.32 [2.02, 2.61]	2.49 [2.12, 3.10]	2.34 [2.06, 2.77]	2.59 [2.18, 3.13]	<0.001
Serum fasting glucose, mmol/L (mean (SD))	5.29 (1.91)	5.08 (2.20)	4.95 (1.54)	5.60 (2.08)	5.27 (1.58)	<0.001
Blood urea nitrogen, mmol/L (median [IQR])	6.40 [4.70, 10.46]	4.75 [3.90, 5.90]	7.40 [5.30, 12.04]	5.62 [4.45, 7.40]	10.10 [6.87, 15.54]	<0.001
Serum creatinine, μmol/L (median [IQR])	106.00 [75.00, 186.00]	74.00 [58.88, 95.93]	132.00 [95.00, 222.50]	88.25 [67.15, 120.10]	184.00 [120.00, 327.55]	<0.001
Uric acid, mmol/L (mean (SD))	437.71 (127.50)	382.40 (110.18)	474.07 (133.59)	414.60 (114.30)	483.84 (127.38)	<0.001
Total cholesterol, mmol/L (mean (SD))	5.17 (2.04)	5.36 (2.25)	4.78 (1.65)	5.54 (2.26)	4.86 (1.70)	<0.001
Triglyceride, mmol/L (median [IQR])	1.46 [1.01, 2.22]	1.22 [0.84, 1.88]	1.43 [1.02, 2.05]	1.58 [1.11, 2.40]	1.54 [1.10, 2.32]	<0.001
HDL-C, mmol/L (mean (SD))	1.18 (0.73)	1.34 (1.30)	1.09 (0.32)	1.20 (0.42)	1.07 (0.37)	<0.001
LDL-C, mmol/L (mean (SD))	3.08 (1.55)	3.26 (1.86)	2.83 (1.24)	3.29 (1.61)	2.85 (1.31)	<0.001
iPTH, pg/mL (median [IQR])	5.76 [3.72, 11.90]	4.46 [3.28, 6.41]	5.86 [3.66, 21.39]	5.20 [3.60, 9.39]	8.23 [4.81, 21.40]	<0.001
Homocysteine, μmol/L (mean (SD))	16.53 (9.08)	10.54 (2.54)	22.03 (9.89)	11.54 (3.14)	23.13 (9.61)	<0.001
eGFR, mL/min/1.73m^2^ (mean (SD))	69.93 (86.78)	101.90 (38.39)	54.64 (36.23)	81.89 (39.41)	42.65 (130.64)	<0.001
Kidney outcomes (%)	380 (14.9)	27 (4.4)	29 (10.2)	96 (12.7)	228 (25.4)	<0.001
median follow-up, mo (SD)	42.17 (0.91)	45.97 (1.28)	36.50 (1.73)	48.00 (1.65)	36.50 (1.73)	–
MACCEs (%)	211 (8.2)	16 (2.6)	19 (6.7)	78 (10.3)	98 (10.9)	<0.001
median follow-up, mo (SD)	36.47 (0.77)	38.77 (1.34)	32.97 (1.58)	41.80 (1.68)	33.13 (0.98)	–

Data were presented by numbers (%) or mean or (SD) median (IQR).

SD, standard deviation; IQR, interquartile range; mo, month; BMI, body mass index; SBP, systolic blood pressure; DBP, diastolic blood pressure; LDL-C, low-density lipoprotein cholesterol; HDL-C, high-density lipoprotein cholesterol; iPTH, intact parathyroid hormone; eGFR, estimated glomerular filtration rate; MACCEs, major adverse cardiac and cerebrovascular events.

The mean homocysteine level was 16.53 μmol/L (SD: 9.08). The prevalence of HHcy was 46.21% (1,182/2,558) among all participants and 54.32% (899/1,655) among those with hypertension. Compared to the normotension with normohomocysteinemia group, individuals with H-type hypertension tended to be older, have a longer disease duration, a higher proportion of male, and were more likely to have diabetes, smoking, and consume alcohol. Additionally, the H-type hypertension group exhibited significantly higher levels of proteinuria, calcium-phosphate product, urea, serum creatinine, uric acid, triglycerides, and intact parathyroid hormone (iPTH), while showing lower levels of hemoglobin, total cholesterol, HDL-C, LDL-C, and eGFR.

### 3.2 H-type hypertension and prognosis

**Figure 2:**
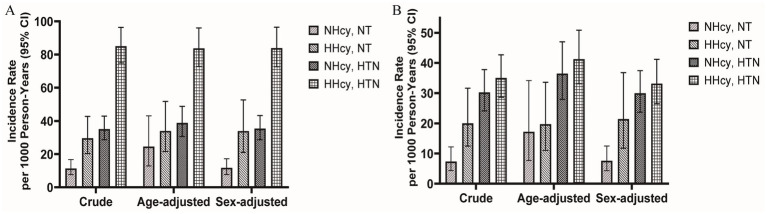
Age-adjusted and sex-adjusted incidence rates per 1,000 person-years with or without hyperhomocysteinemia/hypertension for **(A)** kidney outcomes and **(B)** MACCEs. MACCEs, major adverse cardiac and cerebrovascular events; NT, Normotension; NHcy, Normohomocysteinemia; HHcy, Hyperhomocysteinemia; HTN, Hypertension.

**Figure 3:**
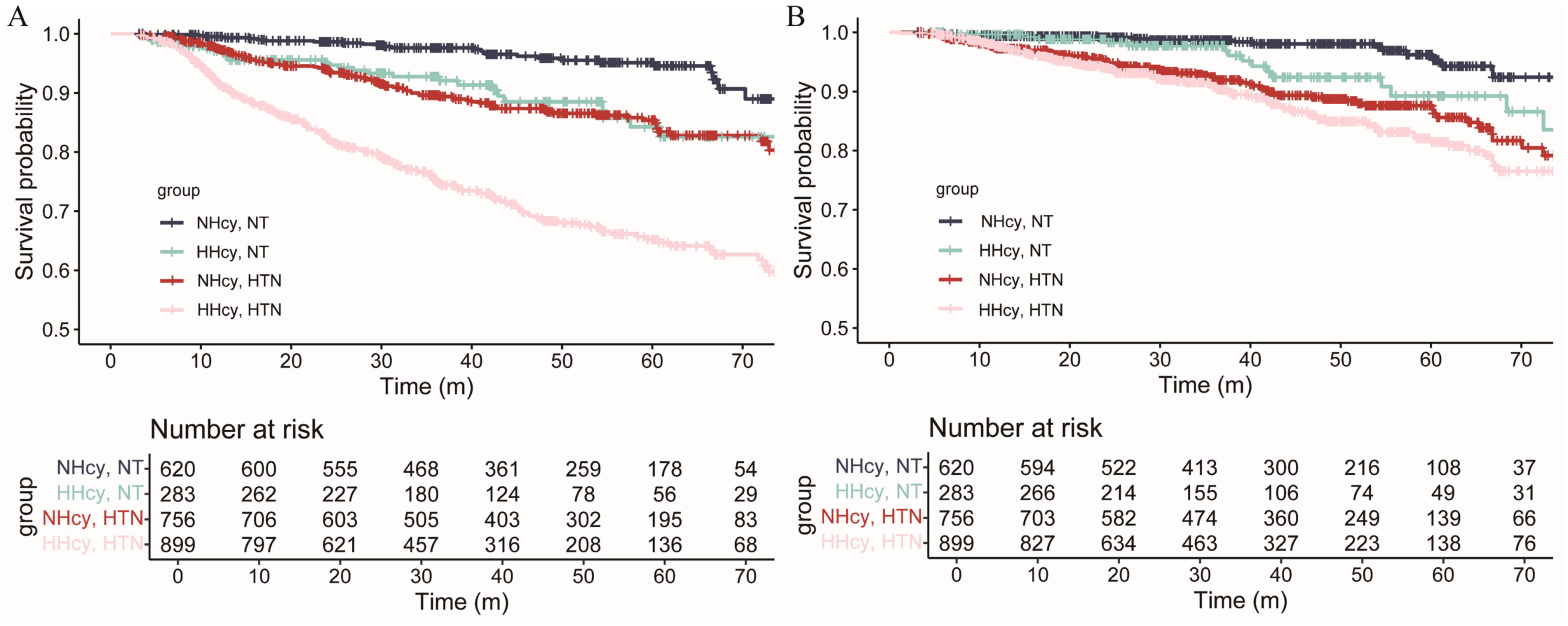
Survival curves of CKD patients with or without hyperhomocysteinemia/hypertension for **(A)** kidney outcomes and **(B)** MACCEs. The risk of kidney outcomes and MACCEs in the H-type hypertension group was significantly elevated (Log-rank test, *p* < 0.001). MACCEs, major adverse cardiac and cerebrovascular events; NT, Normotension; NHcy, Normohomocysteinemia; HHcy, Hyperhomocysteinemia; HTN, Hypertension.

The median follow-up duration for renal outcomes and MACCEs was 42.17 months and 36.47 months, respectively. During follow-up, 380 renal outcomes and 211 MACCEs were recorded. Figure 2 illustrated the age- and sex-adjusted incidence rates per 1,000 person-years. Participants with H-type hypertension had markedly higher rates of renal events (83.71/1,000 person-years vs. 24.50/1,000 person-years) and MACCEs (41.28/1,000 person-years vs. 17.21/1,000 person-years) compared to those without hypertension or hyperhomocysteinemia. Kaplan–Meier survival analysis indicated that H-type hypertension was significantly associated with an elevated risk of both adverse renal outcomes (*p* value of log-rank test <0.001) and MACCEs (*p* value of log-rank test <0.001) (Figure 3). Subsequently, covariates for the multivariable cox regression analysis were selected based on significant associations identified in univariable cox regression analyses (Supplementary Table 2).

Table 2 detailed the unadjusted and adjusted hazard ratios (HRs) and 95% confidence intervals (CIs) for renal outcomes and MACCEs across groups. In the unadjusted model, the risks for renal outcomes and MACCEs in the H-type hypertension group were 7.60 (95% CI 5.10–11.33) and 4.81 (95% CI 2.83–8.17) times respectively, compared to the reference group. For kidney outcomes, the multivariable cox model included hemoglobin, serum creatinine, and intact parathyroid hormone as time-dependent covariates. After adjusting for covariates, H-type hypertension independently increased the risk of kidney outcomes by 312% (HR = 4.12, 95% CI: 2.66–6.37) and MACCEs by 127% (HR = 2.27, 95% CI: 1.28–4.02). The effect values were attenuated but remained significant after full adjustment. Notably, participants in the HHcy/NT group had a significantly elevated risk of renal outcomes (HR = 1.68, 95% CI: 1.00–2.91), whereas no significant difference was found for MACCEs (HR = 1.61, 95% CI: 0.80–3.22). The results of the sensitivity analysis indicate that the findings from the multivariate cox regression model before and after imputation are nearly consistent (Supplementary Table 3). In the adjusted model, every 1 SD rise in homocysteine levels corresponded to a 13% higher risk of renal outcomes (HR = 1.13, 95% CI: 1.03–1.25) among hypertensive CKD patients, while no significant association was found with MACCEs (HR = 1.10, 95% CI: 0.95–1.27). Among normotensive participants, no significant relationship between homocysteine levels and the outcomes was observed.

**Table 2:** Incidence rates per 1,000 person-years and multivariate cox regression analysis of end point events according to hyperhomocysteinemia/hypertension groups.

	Cases/participants	Incidence rate per 1,000 person-years (95% CI)	HR (95% CI)	
			Univariate	Multivariate*
Kidney outcomes				
Groups:				
NHcy, NT	27/620	11.39 (7.67, 16.77)	Ref	Ref
HHcy, NT	29/283	29.64 (20.29, 42.84)	2.63 (1.55, 4.44)	1.68 (1.00, 2.91)
NHcy, HTN	96/756	35.32 (28.85, 43.15)	3.10 (2.02, 4.75)	2.43 (1.56, 3.79)
HHcy, HTN	228/899	85.55 (75.34, 96.97)	7.60 (5.1, 11.33)	4.12 (2.66, 6.37)
Hcy, per 1 SD:				
Total	380/2,558	43.31 (39.20, 47.84)	1.32 (1.26, 1.37)	1.13 (1.03, 1.25)
Hypertension	324/1,655	59.82 (53.73, 66.55)	1.28 (1.21, 1.35)	1.14 (1.03, 1.26)
Normotension	56/903	16.68 (12.74, 21.77)	1.58 (1.26, 1.97)	1.05 (0.72, 1.52)
MACCEs				
Groups:				
NHcy, NT	16/620	7.35 (4.35, 12.19)	Ref	Ref
HHcy, NT	19/283	20.00 (12.43, 31.66)	2.71 (1.39, 5.27)	1.61 (0.80, 3.22)
NHcy, HTN	78/756	30.26 (24.14, 37.81)	4.09 (2.39, 7.00)	2.38 (1.37, 4.14)
HHcy, HTN	98/899	35.02 (28.66, 42.69)	4.81 (2.83, 8.17)	2.27 (1.28, 4.02)
Hcy, per 1 SD:				
Total	211/2,558	24.81 (21.66, 28.40)	1.21 (1.09, 1.34)	1.10 (0.95, 1.27)
Hypertension	176/1,655	32.73 (28.22, 37.93)	1.15 (0.92, 1.29)	1.09 (0.93, 1.28)
Normotension	35/903	11.19 (7.93, 15.71)	1.42 (1.01, 2.01)	1.23 (0.86, 1.76)

Incidence rates per 1,000 person-years was calculated by the indirect method. *Multivariable model adjusted for sex, age, BMI, diabetes, smoking, alcohol consumption, proteinuria, hemoglobin, albumin, calcium-phosphorus product, serum creatinine, uric acid, low-density lipoprotein cholesterol, high-density lipoprotein cholesterol, intact parathyroid hormone. For kidney outcomes, the multivariable model included hemoglobin, serum creatinine, and intact parathyroid hormone as time-dependent covariates.

SD, standard deviation; Hcy, homocysteine; MACCEs, major adverse cardiac and cerebrovascular events; NT, Normotension; NHcy, Normohomocysteinemia; HHcy, Hyperhomocysteinemia; HTN, Hypertension.

### 3.3 Subgroup analysis

To examine the prognostic effects of H-type hypertension in different subgroups, Tables 3, 4 present the results of the subgroup analysis, stratified by demographic characteristics, health status, and medications. Significant interactions after adjusted by Benjamini–Hochberg procedure were found in alcohol consumption (yes vs. no). Regarding MACCEs, significant interactions were observed between diabetes (yes vs. no) with the association between H-type hypertension and MACCEs being stronger in the non-diabetic subgroups. These findings should be interpreted with caution due to imbalanced sample sizes and heterogeneous event rates across subgroups.

**Table 3:** Association between hyperhomocysteinemia/hypertension and risk of kidney outcomes by subgroups.^a^

	Cases/participants	Normotension and normohomocysteinemia	Normotension and hyperhomocysteinemia	Hypertension and normohomocysteinemia	H-type hypertension	Adjusted *p* for interaction
Sex						0.280
Male	231/1,463	Ref	1.16 (0.57, 2.36)	1.42 (0.76, 2.63)	2.83 (1.58, 5.06)	
Female	149/1,095	Ref	2.25 (0.93, 5.41)	3.90 (2.06, 7.40)	4.56 (2.35, 8.87)	
Diabetes						0.265
No	250/1,978	Ref	2.64 (1.36, 5.10)	3.65 (2.12, 6.27)	5.80 (3.36, 9.99)	
Yes	130/580	Ref	0.39 (0.14, 1.10)	0.83 (0.37, 1.85)	1.40 (0.68, 2.90)	
Smoke						0.138
No	262/1,813	Ref	3.04 (1.60, 5.78)	3.63 (2.13, 6.17)	6.21 (3.70, 10.41)	
Yes	118/745	Ref	0.48 (0.18, 1.26)	0.80 (0.37, 1.77)	1.52 (0.76, 3.05)	
Alcohol						0.044
No	281/1,945	Ref	2.54 (1.38, 4.68)	3.16 (1.88, 5.31)	4.85 (2.88, 8.15)	
Yes	146/613	Ref	0.12 (0.02, 0.62)	0.74 (0.31, 1.78)	1.42 (0.64, 3.13)	
BMI						0.598
<24 kg/m^2^	206/1,289	Ref	1.91 (0.93, 3.92)	2.83 (1.55, 5.17)	5.32 (2.97, 9.51)	
24–28 kg/m^2^	117/901	Ref	1.66 (0.63, 4.37)	1.77 (0.79, 3.97)	2.57 (1.15, 5.76)	
≥28 kg/m^2^	57/368	Ref	0.60 (0.06, 5.62)	1.68 (0.54, 5.25)	2.61 (0.84, 8.13)	
Antihypertensive drugs						0.054
No	57/790	Ref	1.19 (0.60, 2.38)	0.34 (0.07, 1.61)	0.99 (0.40, 2.45)	
Yes	323/1,768	Ref	1.50 (0.49, 4.56)	3.63 (1.57, 8.40)	6.32 (2.73, 14.65)	
Folate Tablets						0.598
No	297/2,367	Ref	1.59 (0.89, 2.85)	2.22 (1.39, 3.54)	3.86 (2.43, 6.11)	
Yes	83/191	Ref	4.27 (0.70, 26.01)	7.06 (1.40, 35.52)	6.00 (1.37, 26.25)	
eGFR						0.321
>60 mL/min/1.73 m^2^	69/1,370	Ref	0.48 (0.10, 2.20)	2.46 (1.29, 4.68)	2.22 (0.94, 5.23)	
≤60 mL/min/1.73 m^2^	311/1,188	Ref	0.87 (0.44, 1.71)	1.37 (0.74, 2.54)	1.73 (0.97, 3.08)	

a Cox proportional hazards models adjusted for sex, age, BMI, diabetes, smoking, alcohol consumption, proteinuria, hemoglobin, albumin, calcium-phosphorus product, serum creatinine, uric acid, low-density lipoprotein cholesterol, high-density lipoprotein cholesterol, intact parathyroid hormone. Each of the else groups were adjusted for all covariates expect itself. The *p*-value for the interaction was adjusted using the Benjamini–Hochberg procedure.

BMI, body mass index; eGFR, estimated glomerular filtration rate.

**Table 4:** Association between hyperhomocysteinemia/hypertension and risk of MACCEs by subgroups.^a^

	Cases/participants	Normotension and normohomocysteinemia	Normotension and hyperhomocysteinemia	Hypertension and normohomocysteinemia	H-type hypertension	Adjusted *p* for interaction
Sex						0.774
Male	140/1,463	Ref	1.36 (0.55, 3.38)	2.48 (1.15, 5.31)	2.26 (1.05, 4.88)	
Female	71/1,095	Ref	2.68 (0.87, 8.20)	2.07 (0.91, 4.67)	2.44 (1.00, 6.00)	
Diabetes						0.010
No	121/1,978	Ref	2.73 (1.22, 6.12)	2.64 (1.30, 5.37)	2.67 (1.29, 5.51)	
Yes	90/580	Ref	0.17 (0.02, 1.47)	1.50 (0.62, 3.61)	1.19 (0.46, 3.04)	
Smoke						0.326
No	124/1,813	Ref	2.05 (0.87, 4.87)	2.47 (1.26, 4.84)	1.88 (0.91, 3.89)	
Yes	87/745	Ref	1.18 (0.36, 3.88)	1.94 (0.72, 5.22)	2.58 (0.97, 6.82)	
Alcohol						0.326
No	99/1,945	Ref	2.21 (1.01, 4.84)	2.42 (1.27, 4.61)	2.43 (1.24, 4.78)	
Yes	65/613	Ref	0.46 (0.08, 2.55)	2.07 (0.70, 6.12)	1.94 (0.66, 5.73)	
BMI						0.787
<24 kg/m^2^	98/1,289	Ref	1.74 (0.67, 4.53)	2.71 (1.26, 5.81)	2.54 (1.14, 5.64)	
24–28 kg/m^2^	77/901	Ref	0.70 (0.20, 2.42)	1.80 (0.73, 4.47)	1.36 (0.53, 3.44)	
≥28 kg/m^2^	36/368	Ref	11.47 (1.14, 115.15)	5.87 (0.75, 46.18)	7.15 (0.87, 58.67)	
Antihypertensive drugs						0.326
No	37/790	Ref	2.36 (0.98, 5.68)	2.23 (0.72, 6.93)	0.96 (0.27, 3.42)	
Yes	174/1,768	Ref	1.36 (0.30, 6.22)	3.39 (1.06, 10.88)	3.28 (1.01, 10.65)	
Folate Tablets						0.461
No	187/2,367	Ref	1.70 (0.82, 3.51)	2.40 (1.35, 4.26)	2.53 (1.40, 4.58)	
Yes	24/191	Ref	0.84 (0.06, 11.38)	3.51 (0.32, 38.62)	0.90 (0.10, 8.03)	
eGFR						0.787
>60 mL/min/1.73 m^2^	75/1,370	Ref	1.13 (0.35, 3.58)	2.15 (1.13, 4.10)	1.58 (0.67, 3.75)	
≤60 mL/min/1.73 m^2^	136/1,188	Ref	1.91 (0.54, 6.81)	2.64 (0.79, 8.79)	2.49 (0.77, 8.09)	

a Cox proportional hazards models adjusted for sex, age, BMI, diabetes, smoking, alcohol consumption, proteinuria, hemoglobin, albumin, calcium-phosphorus product, serum creatinine, uric acid, low-density lipoprotein cholesterol, high-density lipoprotein cholesterol, intact parathyroid hormone. Each of the else groups were adjusted for all covariates expect itself. The *p*-value for the interaction was adjusted using the Benjamini–Hochberg procedure.

BMI, body mass index; eGFR, estimated glomerular filtration rate; MACCEs, major adverse cardiac and cerebrovascular events.

### 3.4 Mediation analysis

To calculate the mediated effect of HHcy, mediation analysis was performed. After adjusting for HHcy and other covariates, the hazard ratio for renal outcomes in the hypertensive group was 2.42 (95% CI: 1.80–3.26) compared to the normotensive group, and the HR for MACCEs was 1.82 (95% CI: 1.25–2.66). However, the mediation effect of HHcy on the outcomes was not statistically significant in either the hypertensive or normotensive groups.

### 3.5 PSM and clinical characteristics in CKD patients

Given the limited sample size and potential imbalance between groups, propensity score matching (PSM) was conducted. After PSM, 717 pairs of non-H-type hypertension and H-type hypertension patients, and 517 pairs of NHcy/HTN and HHcy/HTN patients, were included in the analysis (Supplementary Table 5). Histograms of matching pre- and post-propensity scores demonstrated the efficacy of PSM (Supplementary Figure 2). Covariates were balanced across groups following PSM, with standardized mean differences (SMDs) below 0.10 (Supplementary Figure 3). Cox regression models showed that H-type hypertension remained an independent predictor for adverse renal outcomes (HR [95% CI]: H-type hypertension 1.94 [1.50, 2.52]; HHcy/HTN 1.47 [1.10, 1.96]) when compared to the reference. However, no significant difference in the risk of MACCEs was found between the H-type hypertension group and the control group (Supplementary Table 6).

## 4 Discussion

Our study was the first to demonstrate that participants with H-type hypertension experienced a significantly higher incidence of adverse renal outcomes and MACCEs compared to those without hypertension or hyperhomocysteinemia. Over a follow-up period of nearly 4 years, each standard deviation increased in homocysteine levels independently raised the risk of renal outcomes by 13% in CKD patients with hypertension. H-type hypertension was found to increase the risk of renal outcomes and MACCEs by 312 and 127%, respectively, in fully adjusted models. Mediation analysis revealed no significant intermediary effect of HHcy on renal outcomes or MACCEs. Overall, our findings suggested that H-type hypertension was a critical non-traditional risk factor contributing to poor prognosis in CKD patients.

Our research demonstrated a robust association between HHcy and a heightened risk of adverse renal outcomes in CKD patients. In the Chinese population, a high prevalence of methylenetetrahydrofolate reductase (MTHFR) polymorphisms (such as C677T and A1298C) and dietary deficiencies of folate and vitamin B are clinically important in the development of hyperhomocysteinemia and contribute to higher HHcy rates compared to western populations (19, 20). Various studies indicate that more than half of CKD patients exhibit HHcy (6). Among the general population (7), elderly individuals (21), and adults with diabetes (22) or hypertension (23), HHcy is a predictor of CKD onset and further deterioration of renal function. While previous prospective studies exploring the connection between HHcy and renal disease progression have yielded inconsistent results. For example, studies from Samuelsson (24) and Sarna (25) found no significant correlation between total Hcy levels and declining GFR in CKD patients. These discrepancies could be due to small sample sizes, ethnic differences, or varying national folate fortification policies. Nevertheless, HHcy remained a potentially modifiable risk factor, and appropriate folate supplementation should be considered for CKD patients with HHcy, but identifying HHcy in CKD patients remains a top priority.

Our research confirmed that H-type hypertension markedly raised the risk of kidney outcomes in CKD patients, with a higher risk ratio than either HHcy or hypertension alone. Early research has clarified the role of hypertension in advancing CKD. Substantial evidence suggested that HHcy induced atherosclerosis, leading to increased systemic vascular resistance and exacerbating hypertension (26). Xie et al. demonstrated that HHcy significantly elevated the risk of renal function decline in hypertensive patients, highlighting the additive effect of hypertension and HHcy on CKD progression (23). Data from the NHANES survey in the general population also revealed a synergistic interaction between hypertension and HHcy in increasing CKD incidence (27). However, to date, no studies have explicitly assessed whether H-type hypertension exacerbated renal prognosis in CKD patients, which was confirmed by our findings. The CSPPT sub-study showed that enalapril-folate treatment significantly slowed renal deterioration in mild to moderate CKD patients with hypertension compared to enalapril alone (28). A post-hoc analysis of CSPPT by Li et al. revealed that folate-enalapril therapy significantly reduced CKD progression risk in participants with higher baseline B12 levels (29). This finding contrast with results from two other RCTs involving B vitamin supplementation (the DIVINe (30) and HOST (31) studies), which suggested that high doses of B vitamins may accelerate renal function decline. The discrepancies across these studies may arise from differences in B vitamin dosages, baseline eGFR levels, or the fact that the CSPPT was conducted in China, where dietary folate fortification is uncommon, potentially influencing baseline folate and B vitamin levels. Additionally, our research revealed no significant intermediary effect of HHcy on renal outcomes or MACCEs. Potential explanations were as follows. While we adjusted for known confounders, residual confounding (e.g., genetic factors, dietary habits) may lead to violating these assumptions. Hcy levels were measured at baseline, whereas prognosis was assessed over follow-up. Time-dependent changes in Hcy or delayed biological effects may attenuate the observed mediation. The power to detect small-to-moderate mediation effects may have been limited by our cohort size. While HHcy is mechanistically linked to vascular outcomes, its role in hypertension-mediated prognosis may be secondary to other pathways (e.g., oxidative stress, endothelial dysfunction). Our study further emphasized the importance of H-type hypertension as a critical risk factor for CKD patients. Future research should investigate the clinical benefits of simultaneously managing hypertension and HHcy in CKD patients, as well as explore randomized trials to better understand the effects of folate supplementation on CKD progression.

Our study found that H-type hypertension significantly increased the risk of MACCEs in CKD patients, in line with previous research (16). Previous researches have consistently demonstrated that hyperhomocysteinemia was linked to an elevated risk of cardiovascular events, particularly stroke. Early research indicates that the combination of HHcy and hypertension leads to microvascular endothelial dysfunction, early carotid atherosclerosis (32), and increased risk of stroke and cardiovascular events (16, 33). A study on elderly populations revealed that individuals with H-type hypertension had significantly higher odds ratios for stroke incidence and mortality compared to those with either elevated Hcy levels (≥10 μmol/L) or hypertension alone (16). Furthermore, the role of HHcy in cerebrovascular damage was supported by studies showing a dose–response relationship between plasma homocysteine levels and white matter lesions, a marker of cerebrovascular injury, in hypertensive patients (34). This suggested that HHcy may contribute to MACCEs through direct vascular damage. Hcy accumulation in plasma due to impaired metabolic clearance in CKD patients further exacerbates dyslipidemia, insulin resistance (16) and blood pressure fluctuations (35), all of which exacerbate cardiovascular damage in CKD patients. However, the effect of HHcy on MACCEs was not statistically significant, suggesting that hypertension may play a more dominant role. This discrepancy between our results and previous studies could be due to variations in study populations, disease states, and confounding factors. In fact, epidemiological research presents conflicting views on the role of HHcy in the risk of cardiovascular events, particularly in CKD and ESRD patients (13, 14). The heterogeneity within CKD, often reflecting differing inflammation or nutritional status (36), complicates the quantification of cardiovascular risk factors. For example, CKD patients frequently present with hypoalbuminemia, a known predictor of adverse cardiovascular outcomes, and since serum albumin is the primary carrier of circulating Hcy, adjusting for albumin levels may obscure the true impact of Hcy on cardiovascular outcomes. Based on our findings, future research should explore the impact of cardiovascular disease burden in CKD patients with H-type hypertension.

Our data revealed heightened susceptibility to adverse renal and cardiovascular prognostic risk in CKD patients with H-type hypertension. Building on these findings, we propose targeted management strategies. First, folate supplementation should be prioritized in the management of H-type hypertension. Given the central role of HHcy in driving adverse outcomes, daily oral administration of 0.8–1.2 mg folate—a dose supported by the CSPPT trial (29)—is recommended to mitigate HHcy-mediated vascular and renal injury. This intervention may synergize with angiotensin receptor-neprilysin inhibitors (ARNIs). Second, tailored antihypertensive regimens are warranted. For HTH patients with comorbid diabetes or nephropathy, sodium-glucose cotransporter-2 inhibitors (SGLT2i) should be considered first-line (37). Third, individualized risk stratification is critical. Patients presenting with advanced age, reduced eGFR, or dyslipidemia may benefit from intensified monitoring (e.g., quarterly renal function assessments, annual cardiac imaging) and early dual-pathway therapy combining folate and statins to address concurrent vascular calcification risks.

Our study has several limitations. First, as a retrospective study with limited sample size, inherent biases are unavoidable. The stringent exclusion criteria may introduce selection bias, potentially limiting the generalizability of our findings to broader non-dialysis CKD populations. Some variables such as smoking, alcohol consumption, and folate intake were identified by self-report, which may lead to information bias. Second, the lack of intervention in this study prevents the determination of cause–effect relationship. Third, several unadjusted confounding factors, such as MTHFR genetic polymorphisms, nutritional deficiencies, and lifestyle, may have affected the results. Fourth, due to the limited number of participants in the CVD subgroups, we were unable to perform a detailed analysis of specific CVD types. Fifth, the follow-up period may have been too short to capture sufficient outcomes. Lastly, we did not track dynamic changes in Hcy levels during follow-up, and factors such as age, physical activity, smoking, and alcohol consumption could have influenced Hcy levels (38), which could attenuate the observed associations between HTH and adverse outcomes.

In conclusion, our study demonstrates that H-type hypertension is an independent risk factor for both renal progression and MACCEs in CKD patients, even after adjusting for traditional risk factors. These findings highlight the potential clinical value of dual targeting of hypertension and hyperhomocysteinemia in CKD population.

## Data Availability

The original contributions presented in the study are included in the article/Supplementary material, further inquiries can be directed to the corresponding author.
