# Supplementary material for: A retrospective cohort study of H-type hypertension and its influence on the prognostic effect in patients with non-dialysis CKD
Source: Front Nutr. 2025 Mar 25;12:1554663. doi: 10.3389/fnut.2025.1554663 (PMC11975582; doi:10.3389/fnut.2025.1554663)
Supplement: Supplementary file 1 [file Data_Sheet_1.DOCX]

Supplementary Figure 1 DAG illustrating the relationship between outcomes and groups. DAG, directed acyclic graph


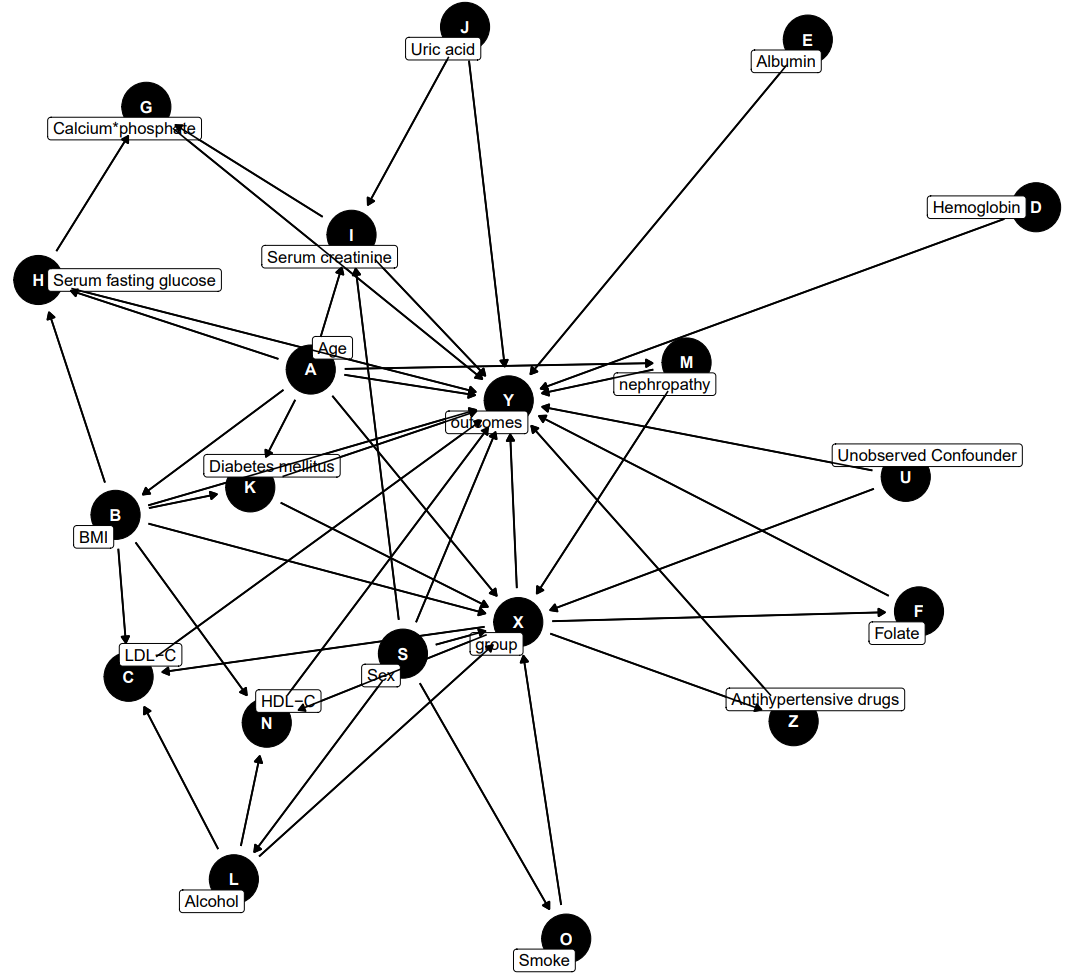


Supplementary Figure 2 Histograms of propensity scores before and after matching. (A) H-type hypertension vs non-H-type hypertension; (B) NHcy/HTN vs HHcy/HTN; NHcy: Normohomocysteinemia; HHcy: Hyperhomocysteinemia; HTN: Hypertension.


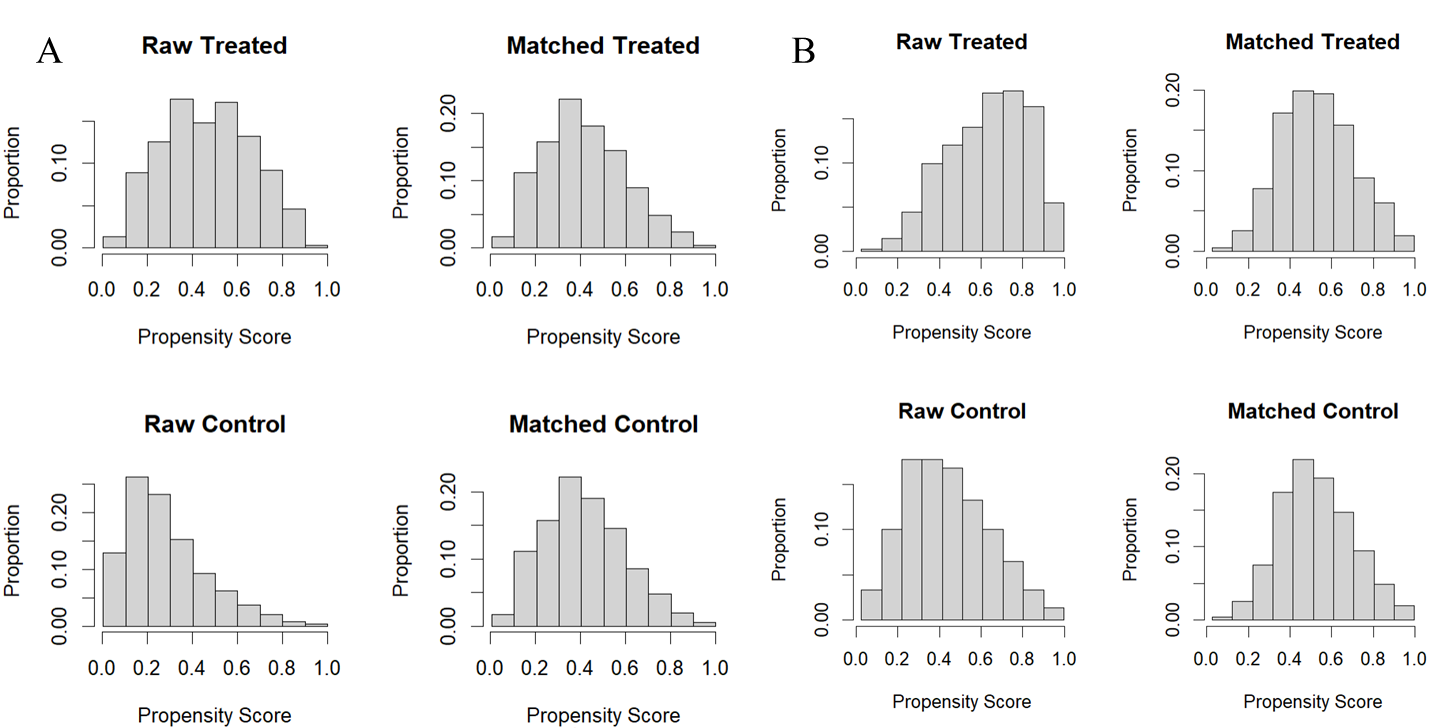


Supplementary Figure 3 Standardized mean difference before and after matching. (A) H-type hypertension vs non-H-type hypertension; (B) NHcy/HTN vs HHcy/HTN; NHcy: Normohomocysteinemia; HHcy: Hyperhomocysteinemia; HTN: Hypertension.


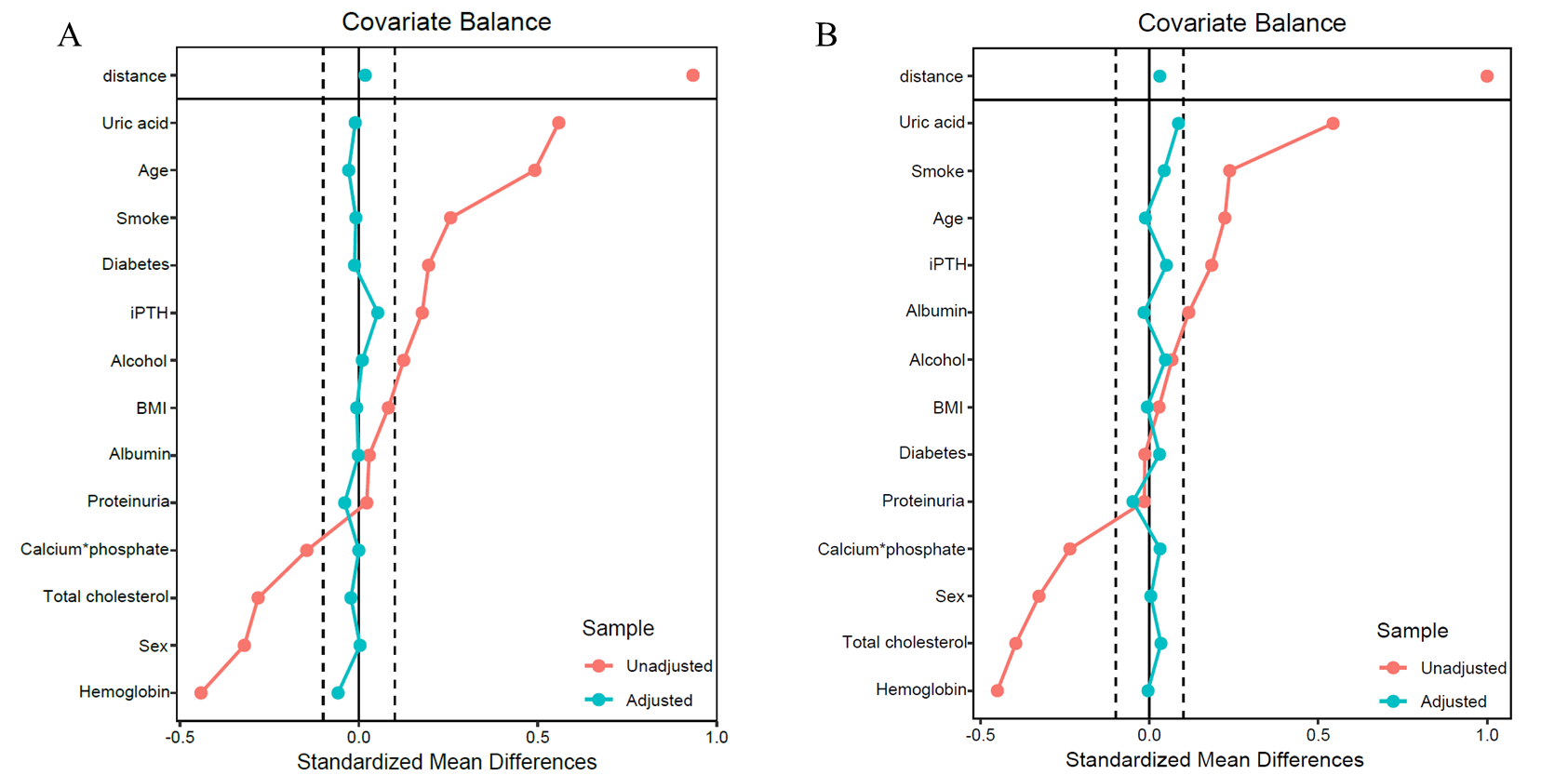


Supplementary Table 1 Missingness report for the retrospective cohort study.

| **Variables** | **Missing number** | **Total number** | **Missing rate (%)** | **Variable type** | **p value*** |
| --- | --- | --- | --- | --- | --- |
| **Proteinuria** | 287 | 2558 | 11.22 | continuous | 0.907 |
| **iPTH** | 251 | 2558 | 9.81 | continuous | 0.552 |
| **Calcium*phosphate** | 115 | 2558 | 4.50 | continuous | 0.982 |
| **Serum fasting glucose** | 82 | 2558 | 3.21 | continuous | 0.876 |
| **BMI** | 65 | 2558 | 2.54 | continuous | 0.907 |
| **Triglyceride** | 34 | 2558 | 1.33 | continuous | 0.598 |
| **HDL-C** | 34 | 2558 | 1.33 | continuous | 0.961 |
| **LDL-C** | 34 | 2558 | 1.33 | continuous | 0.805 |
| **Total cholesterol** | 33 | 2558 | 1.29 | continuous | 0.644 |
| **Albumin** | 17 | 2558 | 0.66 | continuous | 0.961 |
| **Uric acid** | 5 | 2558 | 0.20 | continuous | 0.909 |
| **Blood urea nitrogen** | 4 | 2558 | 0.16 | continuous | 0.965 |
| **Hemoglobin** | 3 | 2558 | 0.12 | continuous | 0.991 |
| **Clinic SBP** | 2 | 2558 | 0.08 | continuous | 0.980 |
| **Clinic DBP** | 2 | 2558 | 0.08 | continuous | 0.988 |
| **Serum creatinine** | 2 | 2558 | 0.08 | continuous | 0.966 |
| **eGFR** | 2 | 2558 | 0.08 | continuous | 0.962 |

*The comparison was assessed using the Wilcoxon rank-sum test. BMI: body mass index; SBP: systolic blood pressure; DBP: diastolic blood pressure; LDL-C: low-density lipoprotein cholesterol; HDL-C: high-density lipoprotein cholesterol; iPTH: intact parathyroid hormone; eGFR: estimated glomerular filtration rate;

Use Little's test to assess for missing completely at random(MCAR), suggesting our data is unlikely to be MCAR

| **Little's test** | **Chi.square** | **(Degrees of freedom used for chisquare)** | **p value** |
| --- | --- | --- | --- |
| **value** | 1494.879 | 1014 | <0.001 |

Supplementary Table 2 Univariate cox regression analysis of end point events.

| **Variable** | **Kidney outcomes** | | **MACCEs** | |
| --- | --- | --- | --- | --- |
|  | **HR (95% CI)** | **p value** | **HR (95% CI)** | **p value** |
| **HHcy, NT** | 2.63 (1.55, 4.44) | <0.001 | 2.71 (1.39, 5.27) | 0.003 |
| **NHcy, HTN** | 3.10 (2.02, 4.75) | <0.001 | 4.09 (2.39, 7.00) | <0.001 |
| **HHcy, HTN** | 7.60 (5.1, 11.33) | <0.001 | 4.81 (2.83, 8.17) | <0.001 |
| **sex** | 0.80 (0.65-0.98) | 0.029 | 0.61 (0.46-0.82) | 0.001 |
| **age, yrs** | 1.02 (1.01-1.03) | <0.001 | 1.05 (1.04-1.06) | <0.001 |
| **diabetes** | 2.23 (1.81-2.76) | <0.001 | 3.18 (2.42-4.18) | <0.001 |
| **smoking** | 1.25 (1.00-1.55) | 0.046 | 2.04 (1.55-2.69) | <0.001 |
| **alcohol consumption** | 1.14 (0.91-1.44) | 0.254 | 1.50 (1.12-2.02) | 0.007 |
| **BMI, kg/m^2^** | 1.00 (0.99-1.01) | 0.918 | 1.00 (1.00-1.01) | 0.114 |
| **proteinuria, g/24 h** | 1.00 (1.00-1.00) | 1.000 | 1.00 (0.99-1.01) | 0.831 |
| **hemoglobin, g/L** | 0.98 (0.97-0.98) | <0.001 | 0.99 (0.99-1.00) | 0.059 |
| **albumin, g/L** | 0.99 (0.98-1.00) | 0.095 | 0.99 (0.97-1.00) | 0.108 |
| **calcium-phosphorus product, mg^2^/dL^2^** | 1.00 (0.99-1.00) | 0.333 | 1.00 (1.00-1.00) | 0.382 |
| **Serum fasting glucose, mmol/L** | 1.05 (1.01-1.09) | 0.012 | 1.08 (1.04-1.12) | <0.001 |
| **Blood urea nitrogen, mmol/L** | 1.06 (1.06-1.07) | <0.001 | 1.02 (1.01-1.04) | 0.001 |
| **serum creatinine, μmol/L** | 1.00 (1.00-1.00) | <0.001 | 1.00 (1.00-1.00) | 0.453 |
| **uric acid, mmol/L** | 1.00 (1.00-1.00) | <0.001 | 1.00 (1.00-1.00) | 0.011 |
| **Total cholesterol, mmol/L** | 0.97 (0.92-1.02) | 0.302 | 0.98 (0.91-1.05) | 0.547 |
| **Triglyceride, mmol/L** | 1.03 (0.99-1.07) | 0.129 | 1.04 (0.99-1.09) | 0.142 |
| **high-density lipoprotein cholesterol, mmol/L** | 0.78 (0.60-1.02) | 0.071 | 0.52 (0.35-0.78) | 0.002 |
| **low-density lipoprotein cholesterol, mmol/L** | 0.91 (0.85-0.99) | 0.022 | 0.97 (0.88-1.07) | 0.543 |
| **intact parathyroid hormone, pg/mL** | 1.00 (1.00-1.00) | <0.001 | 1.00 (1.00-1.00) | 0.070 |
| **eGFR, mL/min/1.73m^2^** | 0.97 (0.97-0.98) | <0.001 | 1.00 (1.00-1.00) | 0.893 |

BMI: body mass index; MACCEs: major adverse cardiac and cerebrovascular events; NT: Normotension; NHcy: Normohomocysteinemia; HHcy: Hyperhomocysteinemia; HTN: Hypertension.

Supplementary Table 3 Sensitivity Analysis: multivariate cox regression analysis of end point events according to Hyperhomocysteinemia/Hypertension groups in the complete case dataset.

|  | cases/participants | HR (95% CI) | |
| --- | --- | --- | --- |
|  |  | Univariate | Multivariate |
| Kidney outcomes |  |  |  |
| Groups: |  |  |  |
| NHcy, NT | 10/252 | Ref | Ref |
| HHcy, NT | 14/151 | 2.45 (1.09, 5.51) | 1.72 (0.75, 3.96) |
| NHcy, HTN | 39/276 | 3.37 (1.68, 6.75) | 2.44 (1.19, 4.99) |
| HHcy, HTN | 88/431 | 6.22 (3.23, 11.96) | 3.23 (1.60, 6.52) |
| Hcy, per 1 SD: |  |  |  |
| Total | 151/1110 | 1.24 (1.13, 1.36) | 0.95 (0.77, 1.16) |
| Hypertension | 127/707 | 1.17 (1.06, 1.31) | 0.96 (0.77, 1.16) |
| Normotension | 24/403 | 1.45 (1.05, 2.01) | 1.07 (0.56, 2.06) |
| MACCEs |  |  |  |
| Groups: |  |  |  |
| NHcy, NT | 5/252 | Ref | Ref |
| HHcy, NT | 11/151 | 3.38 (1.17, 9.74) | 1.91 (0.63, .5.81) |
| NHcy, HTN | 28/276 | 4.29 (1.65, 11.12) | 2.72 (1.03, 7.22) |
| HHcy, HTN | 43/431 | 5.17 (2.05, 13.06) | 2.77 (1.03, 7.46) |
| Hcy, per 1 SD: |  |  |  |
| Total | 87/1110 | 1.15 (0.97, 1.36) | 1.09 (0.86, 1.39) |
| Hypertension | 71/707 | 1.05 (0.85, 1.29) | 1.04 (0.78, 1.39) |
| Normotension | 16/403 | 1.37 (0.88, 2.14) | 1.45 (0.74, 2.85) |

Multivariable model adjusted for sex, age, BMI, diabetes, smoking, alcohol consumption, proteinuria, hemoglobin, albumin, calcium-phosphorus product, serum creatinine, uric acid, low-density lipoprotein cholesterol, high-density lipoprotein cholesterol, intact parathyroid hormone.

SD: standard deviation; Hcy: homocysteine; MACCEs: major adverse cardiac and cerebrovascular events; NT: Normotension; NHcy: Normohomocysteinemia; HHcy: Hyperhomocysteinemia; HTN: Hypertension.

Supplementary Table 4 Mediated effect of hyperhomocysteinemia on the association of hypertension with risk of end point events among CKD ^a^.

|  | HR (95% CI) | | Proportion mediated（%） |
| --- | --- | --- | --- |
|  | Crude ^b^ | Adjusted ^c^ |  |
| Kidney outcomes | 2.55 (1.89, 3.43) | 2.42 (1.80, 3.26) | 5.08 (-8.75, 19.00) |
| MACCEs | 1.83 (1.26, 2.66) | 1.82 (1.25, 2.66) | 0.01 (-5.33, 7.00) |

a Data are HR (95% CI) or %; Mediation analyses were used. The covariates adjusted for in the mediation analyses included sex, age, BMI, diabetes, smoking, alcohol consumption, proteinuria, hemoglobin, albumin, calcium-phosphorus product, serum creatinine, uric acid, total cholesterol, iPTH.

b The crude model included HTN and the covariates.

c The adjusted model included HTN, HHcy, and the covariates.

CKD: chronic kidney disease; MACCEs: major adverse cardiac and cerebrovascular events.

Supplementary Table 5 PSM Baseline Characteristics for H-type hypertension vs non-H-type hypertension and NHcy and HTN vs HHcy and HTN.

|  | non-H-type hypertension (n=717) | H-type hypertension (n=717) | p value | NHcy and HTN (n=517) | HHcy and HTN (n=517) | p value |
| --- | --- | --- | --- | --- | --- | --- |
| Sex: male (%) | 462 (64.4) | 461 (64.3) | 1.000 | 307 (59.4) | 306 (59.2) | 1.000 |
| Age, yrs (mean (SD)) | 51.02 (13.25) | 50.64 (13.07) | 0.588 | 50.24 (13.48) | 50.09 (13.02) | 0.851 |
| Course, mo (median [IQR]) | 7.00 [1.00, 36.00] | 12.00 [1.00, 48.00] | 0.007 | 7.00 [1.00, 36.00] | 10.00 [1.00, 36.00] | 0.258 |
| Composition of nephropathy |  |  | <0.001 |  |  | 0.030 |
| Primary glomerulonephritis (%) | 176 (24.5) | 227 (31.7) |  | 124 (24.0) | 148 (28.6) |  |
| IgA nephropathy (%) | 117 (16.3) | 128 (17.9) |  | 89 (17.2) | 103 (19.9) |  |
| Membranous nephropathy (%) | 66 (9.2) | 22 (3.1) |  | 51 (9.9) | 22 (4.3) |  |
| Minimal Change Disease (%) | 15 (2.1) | 8 (1.1) |  | 15 (2.1) | 8 (1.1) |  |
| Focal Segmental Glomerulosclerosis (%) | 16 (2.2) | 18 (2.5) |  | 11 (2.1) | 13 (2.5) |  |
| Diabetic nephropathy (%) | 89 (12.4) | 101 (14.1) |  | 74 (14.3) | 76 (14.7) |  |
| Hypertensive nephropathy (%) | 53 (7.4) | 81 (11.3) |  | 55 (10.6) | 61 (11.8) |  |
| Hyperuric acid nephropathy (%) | 86 (12.0) | 42 (5.9) |  | 24 (4.6) | 25 (4.8) |  |
| Lupus nephritis (%) | 10 (1.4) | 13 (1.8) |  | 9 (1.7) | 12 (2.3) |  |
| Polycystic Kidney Disease (%) | 11 (1.5) | 22 (3.1) |  | 13 (2.5) | 11 (2.1) |  |
| Obstructive Nephropathy (%) | 22 (3.1) | 14 (2.0) |  | 11 (2.1) | 10 (1.9) |  |
| Diabetes mellitus (%) |  |  |  |  |  |  |
| Smoke (%) | 251 (35.0) | 248 (34.6) | 0.912 | 156 (30.2) | 167 (32.3) | 0.502 |
| Alcohol (%) | 192 (26.8) | 195 (27.2) | 0.905 | 135 (26.1) | 146 (28.2) | 0.485 |
| Antihypertensive drugs | 194 (27.1) | 190 (26.5) | 0.858 | 141 (27.3) | 148 (28.6) | 0.678 |
| Angiotensin‐converting enzyme inhibitors (%) | 30 (4.2) | 34 (4.7) | 0.701 | 46 (8.9) | 23 (4.4) | 0.006 |
| Angiotensin receptor blockers (%) | 235 (32.8) | 240 (33.5) | 0.822 | 225 (43.5) | 178 (34.4) | 0.003 |
| Calcium channel blockers (%) | 250 (34.9) | 446 (62.2) | <0.001 | 296 (57.3) | 309 (59.8) | 0.449 |
| β‐blockers (%) | 107 (14.9) | 186 (25.9) | <0.001 | 125 (24.2) | 120 (23.2) | 0.770 |
| α‐blockers (%) | 39 (5.4) | 79 (11.0) | <0.001 | 40 (7.7) | 52 (10.1) | 0.230 |
| Folate Tablets (%) | 52 (7.3) | 76 (10.6) | 0.033 | 29 (5.6) | 60 (11.6) | 0.001 |
| BMI, kg/m^2^ (median [IQR]) | 24.51 [21.91, 26.83] | 24.45 [22.15, 26.87] | 0.953 | 24.44 [22.15, 27.30] | 24.62 [22.31, 27.10] | 0.869 |
| Clinic SBP, mm Hg (mean (SD)) | 135.19 (21.98) | 148.36 (22.58) | <0.001 | 142.11 (21.19) | 147.53 (23.49) | <0.001 |
| Clinic DBP, mm Hg (mean (SD)) | 83.79 (12.91) | 92.15 (15.19) | <0.001 | 87.69 (14.20) | 91.96 (15.55) | <0.001 |
| Proteinuria, g/24 h (median [IQR]) | 0.59 [0.15, 2.07] | 1.25 [0.41, 2.96] | <0.001 | 0.79 [0.20, 2.38] | 1.21 [0.41, 3.06] | <0.001 |
| Hemoglobin, g/L (mean (SD)) | 122.92 (25.38) | 121.39 (25.83) | 0.256 | 125.17 (24.73) | 125.07 (25.71) | 0.947 |
| Albumin, g/L (mean (SD)) | 37.93 (7.09) | 37.92 (6.63) | 0.976 | 37.70 (7.46) | 37.60 (7.12) | 0.823 |
| Calcium*phosphate, mg^2^/dL^2^ (median [IQR]) | 2.41 [2.09, 2.94] | 2.55 [2.14, 3.05] | 0.012 | 2.35 [2.07, 2.81] | 2.47 [2.11, 3.00] | 0.015 |
| Serum fasting glucose, mmol/L (mean (SD)) | 5.44 (1.85) | 5.22 (1.48) | 0.016 | 5.49 (1.82) | 5.32 (1.61) | 0.116 |
| Blood urea nitrogen, mmol/L (median [IQR]) | 6.40 [4.90, 9.85] | 9.50 [6.52, 14.13] | <0.001 | 5.90 [4.77, 8.09] | 8.47 [6.00, 12.40] | <0.001 |
| Serum creatinine, lmol/L (median [IQR]) | 104.00 [80.00, 168.00] | 168.00 [111.90, 299.10] | <0.001 | 95.00 [76.70, 137.00] | 153.00 [106.00, 251.00] | <0.001 |
| Uric acid, mmol/L (mean (SD)) | 468.15 (121.80) | 466.84 (120.80) | 0.837 | 440.48 (113.75) | 451.45 (123.35) | 0.137 |
| Total cholesterol, mmol/L (mean (SD)) | 5.01 (1.81) | 4.97 (1.72) | 0.685 | 5.10 (1.69) | 5.16 (1.77) | 0.589 |
| Triglyceride, mmol/L (median [IQR]) | 1.53 [1.09, 2.36] | 1.57 [1.12, 2.34] | 0.393 | 1.56 [1.06, 2.36] | 1.67 [1.20, 2.42] | 0.039 |
| HDL-C, mmol/L (mean (SD)) | 1.10 (0.34) | 1.10 (0.39) | 0.703 | 1.16 (0.44) | 1.11 (0.41) | 0.087 |
| LDL-C, mmol/L (mean (SD)) | 2.95 (1.48) | 2.92 (1.32) | 0.654 | 3.00 (1.34) | 3.05 (1.42) | 0.492 |
| iPTH, pg/mL (median [IQR]) | 5.61 [3.65, 12.30] | 7.74 [4.63, 17.81] | <0.001 | 5.52 [3.75, 10.20] | 6.97 [4.43, 14.60] | <0.001 |
| Homocysteine, lmol/L (mean (SD)) | 14.23 (7.13) | 22.85 (9.73) | <0.001 | 11.96 (3.32) | 22.25 (9.50) | <0.001 |
| eGFR, mL/min/1.73m^2^ (mean (SD)) | 67.38 (39.41) | 47.09 (145.64) | <0.001 | 73.51 (37.80) | 46.23 (31.19) | <0.001 |
| Kidney outcomes (%) | 88 (12.3) | 169 (23.6) | <0.001 | 79 (15.3) | 106 (20.5) | 0.035 |
| median follow-up, mo (SD) | 42.17 (1.34) | 36.50 (1.22) | - | 48.13 (2.36) | 36.23 (1.43) | - |
| MACCEs (%) | 66 (9.2) | 71 (9.9) | 0.719 | 61 (11.8) | 55 (10.6) | 0.622 |
| median follow-up, mo (SD) | 36.90 (1.42) | 33.30 (1.01) | - | 42.13 (2.29) | 33.20 (1.28) | - |

Data were presented by numbers (%) or mean or (SD)median (IQR).

NHcy: Normohomocysteinemia; HHcy: Hyperhomocysteinemia; HTN: Hypertension; IQR: interquartile range; SD: standard deviation; IQR: interquartile range; mo: month; BMI: body mass index; SBP: systolic blood pressure; DBP: diastolic blood pressure; LDL-C: low-density lipoprotein cholesterol; HDL-C: high-density lipoprotein cholesterol; iPTH: intact parathyroid hormone; eGFR: estimated glomerular filtration rate; MACCEs: major adverse cardiac and cerebrovascular events.

Supplementary Table 6 Propensity score matched hazard ratios of end point events for H-type hypertension vs non-H-type hypertension and NHcy/HTN vs HHcy/HTN.

|  | HR (95%CI) | |
| --- | --- | --- |
|  | Kidney outcomes | MACCEs |
| non-H-type hypertension | Ref | Ref |
| H-type hypertension | 1.94 (1.50, 2.52) | 1.19 (0.85, 1.67) |
| NHcy and HTN | Ref | Ref |
| HHcy and HTN | 1.47 (1.10, 1.96) | 1.03 (0.71, 1.49) |

NHcy: Normohomocysteinemia; HHcy: Hyperhomocysteinemia; HTN: Hypertension; MACCEs: major adverse cardiac and cerebrovascular events.
